# A Transfer Learning-Based Approach for Lysine Propionylation Prediction

**DOI:** 10.3389/fphys.2021.658633

**Published:** 2021-04-21

**Authors:** Ang Li, Yingwei Deng, Yan Tan, Min Chen

**Affiliations:** School of Computer Science and Technology, Hunan Institute of Technology, Hengyang, China

**Keywords:** propionylation, malonylation, deep learning, transfer learning, recurrent neural network, long short term memory, support machine vector

## Abstract

Lysine propionylation is a newly discovered posttranslational modification (PTM) and plays a key role in the cellular process. Although proteomics techniques was capable of detecting propionylation, large-scale detection was still challenging. To bridge this gap, we presented a transfer learning-based method for computationally predicting propionylation sites. The recurrent neural network-based deep learning model was trained firstly by the malonylation and then fine-tuned by the propionylation. The trained model served as feature extractor where protein sequences as input were translated into numerical vectors. The support vector machine was used as the final classifier. The proposed method reached a matthews correlation coefficient (MCC) of 0.6615 on the 10-fold crossvalidation and 0.3174 on the independent test, outperforming state-of-the-art methods. The enrichment analysis indicated that the propionylation was associated with these GO terms (GO:0016620, GO:0051287, GO:0003735, GO:0006096, and GO:0005737) and with metabolism. We developed a user-friendly online tool for predicting propoinylation sites which is available at http://47.113.117.61/.

## Introduction

No machine is more sophisticated than the cell. This is because there are too many sophisticated mechanisms in the cell, including transcription, gene splicing, translation, and posttranslational modification (PTM). All constituted the sophisticated life. As a key mechanism, PTM increases not only diversities of protein structures and functions but also make regulation more sophisticated. Many studies indicated that aberrant of PTM was always implicated in many human diseases including cancer (Martin et al., 2011; Nakamura et al., 2015; Junqueira et al., 2019). Propionylation, one of more than 400 types of PTM, was firstly discovered in histone in 2007 (Chen et al., 2007), and later in nonhistone (Cheng et al., 2009). The propionylation was a dynamic process where propionyl group was conjugated by some acetyltransferases to substrate proteins which was thus propionylated and could be removed by Sirt1 and Sirt2 (Chen et al., 2007; Leemhuis et al., 2008; Zhang et al., 2008; Cheng et al., 2009). Although it was known that lysine propionylation played a regulating role in the metabolism (Yang et al., 2019) and was a mark of active chromatin (Kebede et al., 2017), many of its unknown functions were still not uncovered.

Identifying propionylation sites was crucial to further explore functions of propionylated proteins. The mass spectrometry has been developed to detect propionylation sites in the past decades and obtained vast achievements (Chen et al., 2007). However, this technique was time consuming and labor intensive. Another alternative was computational methods which learned a model from the known data and then gave the predictions for unknown data. The process was similar with learning of human. In the past 30 years, more than 100 computational methods or tools have been developed for predicting PTM sites (Huang and Zeng, 2016; Zhou et al., 2016; Ai et al., 2017; Wei et al., 2017, 2019; Xiang et al., 2017; Chen et al., 2018; de Brevern et al., 2018; Ning et al., 2018, 2019; Xie et al., 2018; Huang et al., 2019, 2020; Luo et al., 2019; Malebary et al., 2019; Wang et al., 2019; Lv et al., 2020; Qian et al., 2020; Thapa et al., 2020). For example, Malebary et al. (2019) proposed a computational model for lysine crotonylation prediction by integrating various position and composition relative features along with statistical moments, and reached the average accuracy of 0.9917 in the experimental dataset. Chen et al. (2018) presented a computational tool named ProAcePred to predict prokaryote lysine acetylation sites by extracting sequence-based, physicochemical property and evolutionary information features. Wei et al. (2017, 2019) used sequence-based information to build computational models for predicting phosphorylation sites and protein methylation sites, respectively. Although propionylation was a newly discovered PTM, there still were two computational methods developed to detect propoinylation sites. One was that the biased support vector machine (SVM) model (Ju and He, 2017) which incorporated four different sequence features into Chou’s pseudo-amino acid composition. Another was the PropSeek which was also a SVM model and which exploited evolutionary information, sequenced-derived information, predicted structural information, and feature annotations (Wang et al., 2017). Advance in deep learning techniques could accelerate development of propionylation prediction. A well-known example was that the AlphaFold, a deep-learning-based method, accurately determined a protein’s 3D shape from its amino-acid sequence (Callaway, 2020). The detection of protein structure especially in more than two dimensions was one of biology’s grandest challenges and to date no better technique can solve this issue. In this paper, we attempted to build a deep learning model to predict propionylation sites. However, the accumulated propionylation data was too small to better train deep learning model. Lysine propionylation has *in situ* crosstalk with lysine malonylation. Wang et al. (2017) statistically compared 1,471 propionylation sites in 605 proteins with the dataset of 1,745 malonylation sites in 595 proteins and found that 600 (40.8%) of 1,471 propionylation sites are overlapped with malonylation. What is more, the number of malonylation was much more than that of propionylation sites. Inspired by this, we proposed a transfer learning method for predicting propionylation sites. We firstly constructed a recurrent neural network (RNN)-based deep learning model, which was trained by the malonylation data. The model was then fine tuned by the propionylation data. The model served as feature extractor. Finally, the SVM-based classifier was trained to discriminate propionylation from nonpropionylation.

## Data

All lysine propionylation sites were both from the protein lysine modifications database (PLMD) (Xu et al., 2017) and Uniprot database (UniProt Consortium, 2018). The PLMD was devoted to collect lysine modification, currently hosting 284,780 modification events in 53,501 proteins for 20 types of lysine modification such as ubiquitination, methylation, and sumoylation. The Uniprot was a comprehensive database of protein sequence and function annotation. We firstly downloaded 192 proteins containing 413 propionyllysine sites from the PLMD http://plmd.biocuckoo.org/download.php. We then retrieved 18 propionylation proteins from the Uniprot database. After merging two dataset of proteins and removing repeated proteins, we obtained 207 unique proteins. Functions of protein including protein modification would rely more or less on homology. To reduce or remove influences of homology on the proposed method, we applied the sequence clustering software CD-HIT (Li and Godzik, 2006) to perform sequence clustering. The sequence identity was set to 0.7. Finally, we obtained 189 proteins as experimental data, of which sequence similarities between any two was less than 0.7. We selected randomly 4/5 of 189 proteins (151) as positive training samples which containing 304 sites, the remaining (38) as positive testing ones containing 104 sites. Lysine sites largely outnumbered lysine propionylation sites, so positive and negative samples were unbalanced, i.e., negative samples extremely outnumbered positive ones. The unbalance between positive and negative samples would cause the trained model to prefer to negative samples. Therefore, we randomly selected sites of lysine which does not undergo PTM from these proteins as negative samples at a ratio of positive to negative 1:1. The training set consisted of 304 positive and 304 negative lysine sites, while the testing set of 104 positive and 104 negative lysine sites. All the positive and the negative sites are listed in the Supplementary Material.

We also downloaded 3,429 malonylated proteins containing 9,584 malonylation sites. Similarly, we randomly chose the same number of lysine sites as nonmalonylation sites, These lysine sites did not undergo malonylation events as negative samples. Therefore, the malonylation set contained 9,584 malonylation sites and 9,584 nonmalonylation lysine sites.

## Materials and Methods

**FIGURE 1:**
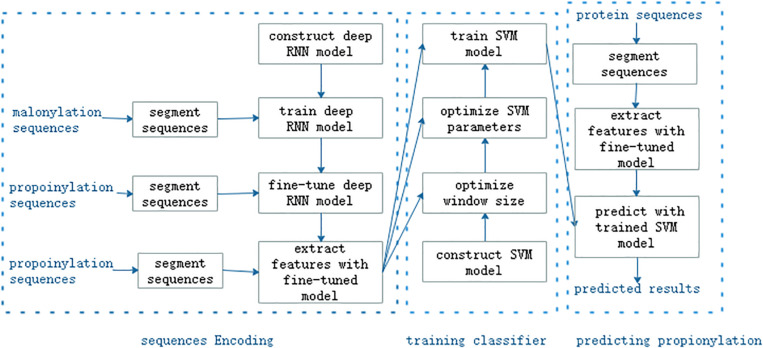
The workflow of the proposed method.

As shown in Figure 1, the proposed method consisted of three main steps: feature encoding, training classifier, and predicting propionylation, or eight modules: segmenting sequences, constructing a deep RNN model, training the deep RNN model, extracting features, constructing SVM model, optimizing the window size and the super-parameters in the SVM model, training the SVM model, and predicting propionylation with trained SVM model. We used the malonylation dataset to train the RNN model and then fine tuned the trained model by the training set of propionylation data. Propionylation sequences were inputted into the fine-tuned and trained deep RNN model and the outputs in its last second layer were viewed as features of the propionylation sequences. The subsequent workflow was the same as the common machine learning method.

### Segmenting Sequences

**FIGURE 2:**
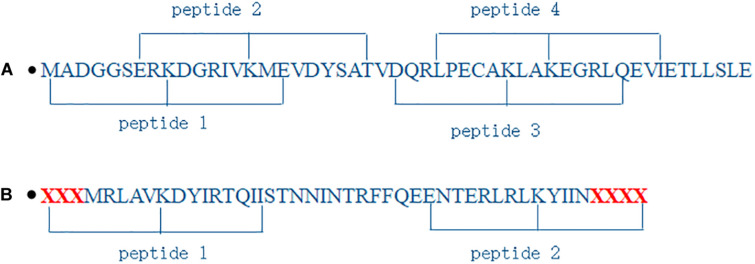
Illustration of segmenting protein sequences. **(A)** is normal segment; **(B)** is segment when the number of residues is less than 8.

As shown in Figure 2A, protein sequences were segmented into peptides where lysine was the center and *n* residues were located in its downstream and upstream, respectively. If the number of residues in the downstream or the upstream was less than *n*, the corresponding number of character *X* were supplemented, as shown in Figure 2B. The peptides were a window of residues in fixed size (2×*n* + 1). We obtained 816 peptides, and 9,584 + 9,584 = 19,168 peptides for propionylation dataset and for malonylation dataset above, respectively.

### Deep RNN Model

**FIGURE 3:**
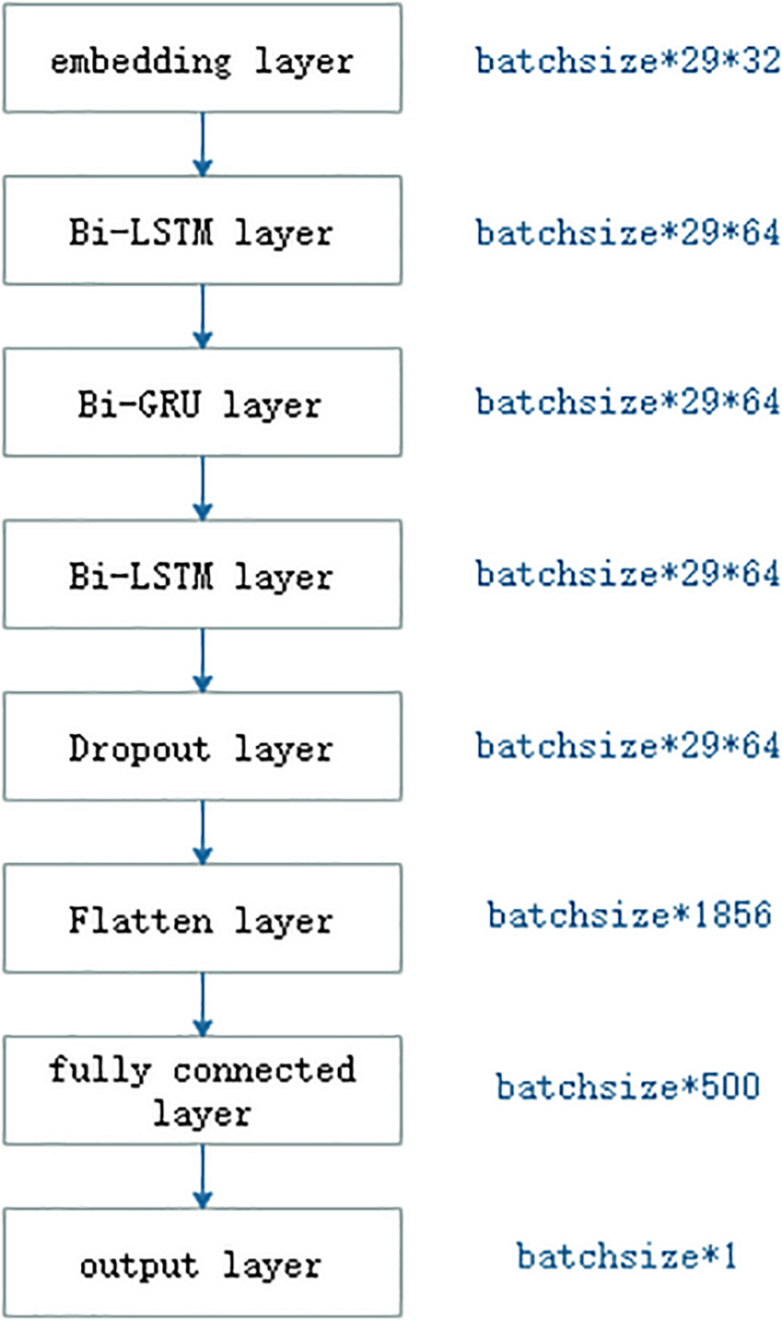
The RNN-based deep learning model.

As shown in Figure 3, the deep RNN model was made up of one embedding, two long short-term memory (LSTM), one Gated Recurrent Unit (GRU), one dropout, one flatten, one fully connected, and one output layer. The embedding layer translated integer indices of amino acid characters into embedding vectors. In general, the embedding layer was regarded as a bridge from text to numerical vector in field of natural language process. The LSTM (Hochreiter and Schmidhuber, 1997) was a RNN (Pearlmutter, 1989; Giles et al., 1994). The RNN shared network weights where output at current step not only depended on the input at current step but also on output at previous steps. Due to its effect and efficiency, the RNN has widely been applied in the field of sequence analysis or time-series analysis. The RNN could not remember information about previous inputs which was away from the current input. The LSTM was one of better solutions to it. The typical LSTM included three gates: forget gate, input gate, and output gate. The forget gate was to forget some past information selected, and the input gate was to remember some current information. All three gates adopted the sigmoid as the activation function, whose output ranged from 0 to 1. The output was 0, meaning that no information was passed and 1 meant all information was passed. The LSTM also included a candidate memory cell which fused current and past memories. The GRU was a variant of the LSTM. Compared with the LSTM, the GRU included only two gates: reset gate and update gate, dropping the candidate memory cell. The reset gate was to determine which past information to be forgotten, and the update gate to drop some past information and to add some new information. The number of operations in the GRU was less than that that in the LSTM, so the GRU was computed faster than the LSTM. For the purpose of detecting bidirectional semantic information, we used the bidirectional LSTM and the bidirectional GRU.

Deep learning model would cause overfitting and be time consuming. Hinton et al. (2012) proposed a dropout operation as a solution to prevent neural networks from overfitting. The dropout operation was to drop some neurons whose weights were not updated during training at a certain rate of dropout, while all the neurons were used during testing. Since the dropout was created, it is becoming a more prevalent trick in the deep learning models (Srivastava et al., 2014).

Flatten layer was a bridge between the LSTM layer and fully connected layer, and its aim was only to transform the shape of input so that it could be connected to the subsequent fully connected layer. The fully connected layer corresponded to the hidden layer in the multiple layer perception. The number of neurons in the output layer was responsible for the number of class labels.

### Support Vector Machine

The SVM proposed by Vapnik et al. (Boser et al., 1992; Cortes et al. 1995; Vapnik et al. 1998) is a statistical learning algorithm. Due to mathematically theoretical foundation, the SVM has been applied to a wide range of fields from handwritten digit recognition (Matic et al., 1993), text categorization (Joachims, 1999), face images detection (Osuna et al., 1997), to protein/gene structure or function prediction (Caragea et al., 2007; Plewczynski et al., 2008; Li et al., 2009; Pugalenthi et al., 2010; Li et al., 2011; Sun et al., 2015; Ning et al., 2018). Take, for example, a binary classification with the *n* training samples {(*x*_*i*_,*y*_*i*_)|*i* = 1,2,…,*n*} where *y*_*i*_ ∈ {1,−1}. The SVM aimed to find a hyperplane *f*(*x*) = *wx* + *b* to separate samples with positive label 1 from ones with label −1. That is to say, the hyperplane made positive samples satisfy *f*(*x*) = *wx* + *b* > 0 and negative ones satisfy *f*(*x*) = *wx* + *b* < 0. In fact, there would be many hyperplane meeting the requirement above. The SVM was to find such a hyperplane that maximizes the separating margin. This question was modeled as minimizing the following formulas:

(1) $$L\,\left(w\right)=\frac{1}{2}\,w^{T}\,w,$$

subject to the constraints:

(2) $$y_{i}\,\left(w\,x_{i}+b\right)\geq 1,\,i\,=1,2,3,\ldots ,n.$$

In the real world, the training samples were not always completely separable by any hyperplane. That is to say, there were some samples which were separated as another category. To address this issue, the SVM introduced the slack variables ξ_*i*_. The objective function (1) was rewrote as

(3) $$L\,\left(w,\xi_{i}\right)=\frac{1}{2}\,w^{T}\,w+C\,\sum_{i=1}^{n}\xi_{i},$$

where *C* was called penalty factor, a user-specified hyper-parameter, while the constraint (2) was rewrote as

(4) $$y_{i}\,\left(w\,x_{i}+b\right)\geq 1-\xi_{i},i=1,2,3,1/4,n,\xi_{i}\geq 0$$

The objective function was composed of the structural risk (the first term in Eq. 3) and empirical risk (the second term in Eq. 3). The penalty factor controlled trade-off between two risks. Another superiority of the SVM was that it absorbed the kernel function. There existed a case that samples could be not discriminable in the low-dimensional space, but they became discriminable. The SVM firstly exploited the kernel function to transform these undistinguishable samples from low-dimensional into high-dimensional shape, and then found a high-dimensional hyperplane to separate them, which was expressed by

(5) $$F\,\left(x\right)=w^{T}\,\text{f}\,\left(x\right)+b$$

where ϕ(*x*) was a kernel function. There are more than ten kernel functions such as linear kernel $\phi \,\left(x_{i},x_{j}\right)=x_{i}^{T}\,x_{j}$, polynomial kernel $\phi \,\left(x_{i},x_{j}\right)={\left(a\,x_{i}^{T}\,x_{j}+c\right)}^{d}$, Gaussian Kernel $\phi \,\left(x_{i},x_{j}\right)=\exp\left(-\frac{{\left|x_{i}-x_{j}\right|}^{2}}{2\,\sigma^{2}}\right),$ etc. The corresponding constraint were updated as

(6) $$y_{i}\,\left(w\,\phi \,\left(x_{i}\right)+b\right)\geq 1-\xi_{i},i=1,2,3,\ldots ,n,\xi_{i}\geq 0$$

The SVM was soluble by the dual theory and the Lagrange optimization algorithm. Readers can refer to the relevant scientific references.

## Crossvalidation and Metrics

In the case of regression or classification question, there are generally four types of validations: hold-out validation, *k*-fold crossvalidation, leave-one-out, and independent test. In the hold-out validation, the training set was splitted into two parts: one for training and another for validation. In the *k*-fold cross validation, the training set was divided into *k* parts. Each part was tested by the model trained over other *k* − 1 parts. Leave-one-out was an extreme cross validation, where the number of samples is equal to *k*. We used 10-fold cross validation and independent test to examine the proposed method.

To quantitatively compare performance of methods, the following metrics: sensitivity (SN), specificity (SP), accuracy (ACC), and Matthews correlation coefficient (MCC), were used, which were computed by

$$\text{SN}=\frac{\text{TP}}{\text{TP}+\text{FN}}$$

$$\text{SP}=\frac{\text{TN}}{\text{FP}+\text{TN}}$$

$$\text{ACC}=\frac{\text{TP}+\text{TN}}{\text{TP}+\text{FN}+\text{FP}+\text{TN}}$$

$$\text{MCC}=\frac{\mathrm{TP}\times \text{TN}-\mathrm{FP}\times \text{FN}}{\sqrt{\left(\text{TP}+\text{FN}\right)\,\left(\text{TP}+\text{FP}\right)\,\left(\text{TN}+\text{FN}\right)\,\left(\text{TN}+\text{FP}\right)}}$$

In the equations above, TP is the number of the true positive samples, TN the number of the true negative samples, FN the number of false-negative samples, and FP the number of false-positive samples. SN, SP, and ACC ranges from 0 to 1, 0 meaning completely wrong and 1 completely correct. For example, SN = 0 implied that all the positive samples were predicted as negative ones. MCC ranges from −1 to 1, 1 meaning perfect prediction, 0 random prediction, and −1 the prediction completely opposite to the true.

The receiver operating characteristic (ROC) curve was used to depict performance, which plotted true positive rate against false positive rate under various thresholds. The area under the ROC curve (AUC) was used to quantitively assess the performance. The AUC ranged from 0 to 1, 0.5 meaning random guess and 1 perfect performance.

## Results

### Parameter Optimization

The size of peptide window was generally set to one of the interval [21, 41]. We conducted 10-fold crossvalidations over the training set to search for better window size. The performances under various window size were listed in Table 1. The crossvalidation of window size 29 obtained the better performance. Therefore, we set window size to 29 in the subsequent experiments. We also optimized super parameters in the SVM classifier, i.e., *C*, kernel, and gamma. We searched combination space of *C* = [0.5, 1, 1.5, 2, 2.5, 3, 10, 100, 1,000], kernel = [“linear,” “poly,” “rbf”], and gamma = [“scale,” “auto”]. Table 2 shows the best 15 combinations. The best performance was SN = 0.8454, SP = 0.8158, ACC = 0.8306, and MCC = 0.6615, slightly better than previous, and the corresponding parameter was that *C* = 1, kernel = rbf, and gamma = scale. The predictive performance in the testing set was a SN of 0.6731, a SP 0.6442, an ACC of 0.6587, and a MCC of 0.3174.

**TABLE 1:** Performance of various window size in the 10-fold crossvalidation.

Size	SN	SP	ACC	MCC
21	0.6579	0.7862	0.7220	0.4478
23	0.7631	0.8421	0.8026	0.6072
25	0.7697	0.8553	0.8125	0.6273
27	0.7533	0.7763	0.7648	0.5297
29	**0.8355**	0.8158	**0.8257**	**0.6514**
31	0.7697	0.8059	0.7878	0.5760
33	0.7928	**0.8553**	0.8240	0.6493
35	0.7664	0.7796	0.7730	0.5461
37	0.7500	0.7697	0.7599	0.5198
39	0.7467	0.7336	0.7401	0.4803
41	0.7697	0.7434	0.7566	0.5133

*Bold values mean the best in the column.*

**TABLE 2:** The best 15 combinations in the searching space.

*C*	Gamma	Kernel	Average accuracy
1	Scale	rbf	0.8389
1	Auto	rbf	0.8389
0.5	Scale	rbf	0.8356
0.5	Auto	rbf	0.8356
1.5	Scale	rbf	0.8307
1.5	Auto	rbf	0.8307
2	Scale	rbf	0.8258
2	Auto	rbf	0.8241
2.5	Scale	rbf	0.8143
2.5	Auto	rbf	0.8143
3	Auto	rbf	0.8093
3	Scale	rbf	0.8093
0.5	Auto	Sigmoid	0.7960
0.5	Scale	Sigmoid	0.7960
1	Auto	Sigmoid	0.7664

### Comparison With Other Methods

To the best of my knowledge, there were two computational methods for propionylation prediction. One was the PropPred (Ju and He, 2017) and another was the PropSeek (Wang et al., 2017). However, to date, these two webservers stopped work. The performance of the PropPred with 250 optimal features and a window size of 25 residues in the 10-fold crossvalidation was a SN of 0.7003, a SP 0.7561, an ACC of 0.7502, and a MCC of 0.3085, inferior to that of the proposed method. The performance of the PropPred in the testing set was a SN of 0.6604, a SP of 0.7504, an ACC of 0.7431, and a MCC of 0.2495, inferior to that of the proposed method in terms of SN and MCC. It must be pointed out that the training and the testing set used by two methods were different. To perform fair comparison, we implemented the PropProd with the 250 optimal features and a window size of 25 residues. Both performances of 10-fold crossvalidation on the training set and of independent test on the testing set are listed in Table 3. Obviously, the proposed method outperformed the PropPred. We also compared the presented method with the deep RNN model. The performance of the deep RNN model over the testing set obtained a SN of 0.5962, a SP of 0.6731, an ACC of 0.6346, and a MCC of 0.2700. The presented method outperformed the deep RNN model.

**TABLE 3:** Performances of the PropPred method.

	SN	SP	ACC	MCC
10-fold	0.7928	0.7599	0.7763	0.5529
Independent	0.4904	0.6442	0.5673	0.1362

**FIGURE 4:**
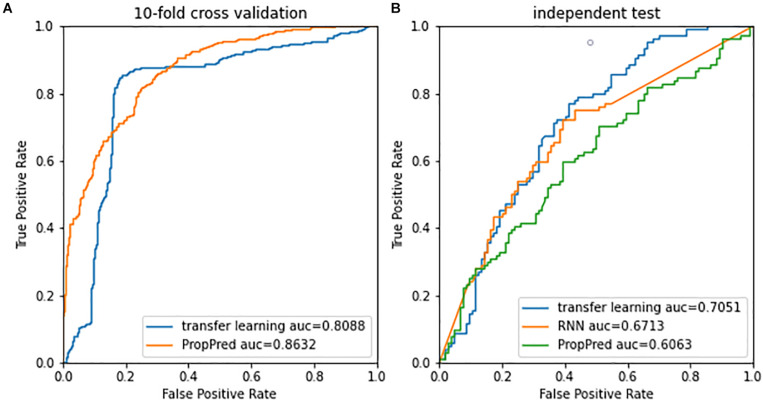
Receiver operating characteristic curves of **(A)** 10-fold cross validation and **(B)** independent test.

Figure 4A shows performances of 10-fold crossvalidation for the presented method and the PropPred. Although the AUC of the presented method was inferior to that of the PropPred, the best performance at the most up-left was better than that of that of the PropPred. In the independent test (Figure 4B), the presented method outperformed the PropPred and the deep RNN method. Obviously, the presented method occupied advantage of the deep learning and avoided artificial design of feature extraction.

### Functional Analysis

We used the DAVID web application (Huang da et al., 2009) for functional analysis which included a comprehensive set of functional annotation tools to uncover and understand biological meaning behind studied genes. Firstly, we exploited the gene functional classification tool in the DAVID to cluster 183 proteins from Thermus thermophilus HB8. As shown in Table 4, only 29 proteins clustered into four similar function groups, while other proteins showed no similarity of functions. The proteins leucyl-tRNA synthetase (leuS) and the protein histidyl-tRNA synthetase (hisS) appeared simultaneously in two groups. We also used the function annotation tool in the DAVID perform enrichment of GO and KEGG pathway. Because 183 of 207 proteins were from Thermus thermophilus HB8, genes of Thermus thermophilus HB8 were used as background. Under the condition of ease less than or equal to 0.01, the enriched GO terms of molecular function were GO:0016620 (oxidoreductase activity, acting on the aldehyde or oxo group of donors, NAD or NADP as acceptor), GO:0051287 (NAD binding), and GO:0003735 (structural constituent of ribosome). The enriched GO terms of biological process and cellular component was GO:0006096 (glycolytic process) and GO:0005737 (cytoplasm), respectively, as shown in Table 5. The enriched pathways are listed in Table 6. In the nine enriched pathways, four was related to metabolism, and two to biosynthesis, implying involvement roles of the propionylation in the metabolism. Some researchers reported that lysine propionylation was involved in metabolism (Okanishi et al., 2014, 2017; Yang et al., 2019).

**TABLE 4:** Function groups of proteins.

UNIPROT _ACCESSION	Gene name	Enrichment score
Q5SIR5	Ribose-5-phosphate isomerase A (TTHA1299)	3.8325
Q5SIC8	Fructose 1,6-bisphosphatase II (glpX)	
Q5SM35	Transketolase (TTHA0108)	
Q5SHF7	Fructose-1,6-bisphosphate aldolase (TTHA1773)	
Q5SM37	Ribulose-phosphate 3-epimerase (TTHA0106)	
Q5SLJ4	Glucokinase (TTHA0299)	
Q5SJM8	Hypothetical protein (TTHA0980)	
**P56194**	Histidyl-tRNA synthetase (hisS)	3.2378
**Q5SLY2**	Leucyl-tRNA synthetase (leuS)	
Q5SJX7	Seryl-tRNA synthetase (TTHA0875)	
P56881	Threonyl-tRNA synthetase (thrS)	
P56206	Glycyl-tRNA synthetase (TTHA0543)	
P56690	Isoleucyl-tRNA synthetase (ileS)	2.5835
P23395	Methionyl-tRNA synthetase (TTHA1298)	
**P56194**	Histidyl-tRNA synthetase (hisS)	
**Q5SLY2**	Leucyl-tRNA synthetase (leuS)	
Q5SJ45	Valyl-tRNA synthetase (valS)	
Q5SIH0	Tyrosyl-tRNA synthetase (TTHA1399)	
P80380	30S ribosomal protein S20 (rpsT)	1.8414
Q5SHQ2	30S ribosomal protein S8 (rpsH)	
Q5SHP6	50S ribosomal protein L29 (TTHA1684)	
Q5SHQ5	30S ribosomal protein S5 (rpsE)	
Q5SLP7	50S ribosomal protein L1 (rplA)	
Q5SHQ0	50S ribosomal protein L5 (rplE)	
P80377	30S ribosomal protein S13 (rpsM)	
Q5SHN3	30S ribosomal protein S12 (rpsL)	
P35871	50S ribosomal protein L33 (rpmG)	
Q8VVE2	50S ribosomal protein L7/L12 (rplL)	
Q5SLY1	30S ribosomal protein S1 (rpsA)	
P17291	30S ribosomal protein S7 (TTHA1696)	
Q9Z9H5	50S ribosomal protein L17 (rplQ)	

*Bold values mean repeat.*

**TABLE 5:** Significantly enriched GO terms.

Category	Term	Count	*P* value
GOTERM_CC_DIRECT	GO:0005737 cytoplasm	38	1.07E-05
GOTERM_MF_DIRECT	GO:0016620 oxidoreductase activity, acting on the aldehyde or oxo group of donors, NAD or NADP as acceptor	5	1.91E-03
GOTERM_BP_DIRECT	GO:0006096 glycolytic process	6	3.07E-03
GOTERM_MF_DIRECT	GO:0051287 NAD binding	8	3.09E-03
GOTERM_MF_DIRECT	GO:0003735 structural constituent of ribosome	13	9.83E-03

**TABLE 6:** Significant KEGG pathways.

Term	Count	*P* value
ttj01200:Carbon metabolism	35	2.18E-09
ttj01120:Microbial metabolism in diverse environments	44	1.49E-07
ttj01130:Biosynthesis of antibiotics	43	4.16E-06
ttj00010:Glycolysis/gluconeogenesis	15	3.92E-05
ttj00020:Citrate cycle (TCA cycle)	12	1.52E-04
ttj00620:Pyruvate metabolism	14	5.84E-04
ttj00710:Carbon fixation in photosynthetic organisms	8	5.95E-04
ttj01110:Biosynthesis of secondary metabolites	50	7.43E-04
ttj01100:Metabolic pathways	85	8.13E-04

## Conclusion

We presented a transfer learning-based method and an online webserver^1^ for computationally predicting propionylation. The method took advantage of crosstalk between propionylation and malonylation. The advantage of the method was to avoid artificially designing features. Statistical enrichment analysis implied that propoinylation was associated with metabolism.

## Data Availability Statement

The original contributions presented in the study are included in the article/Supplementary Material, further inquiries can be directed to the corresponding authors.

## Author Contributions

AL and YD: conceptualization, funding acquisition, and writing – original draft. YT: data curation, formal analysis, and software. AL, MC, and YD: methodology, validation, writing – review, and editing. All authors contributed to the article and approved the submitted version.

## Conflict of Interest

The authors declare that the research was conducted in the absence of any commercial or financial relationships that could be construed as a potential conflict of interest.

## Footnotes

1 http://47.113.117.61/
